# A Rare Case of PL-7-Associated Immune-Mediated Necrotizing Myopathy With Isolated Dysphagia as the Presenting Symptom

**DOI:** 10.7759/cureus.37215

**Published:** 2023-04-06

**Authors:** Tahir Khan, Aleeya Shareef, Mohammad Shahid, Ehsan Shabbir, Mustafa Musleh

**Affiliations:** 1 Internal Medicine, Premier Miami Valley Hospital, Dayton, USA; 2 Internal Medicine, Wright State University Boonshoft School of Medicine, Dayton, USA; 3 Gastroenterology, Premier Miami Valley Hospital, Dayton, USA

**Keywords:** pl 7 antibodies, anti pl7, proximal muscle weakness, necrotizing autoimmune myopathy (nam), inflammatory myopathy, dysphagia, immune mediated necrotizing myopathy (imnm)

## Abstract

Immune-mediated necrotizing myopathy (IMNM) is a rare, progressive disease that accounts for about 19% of all inflammatory myopathies. Dysphagia occurs in about 20%-30% of IMNM patients. This case results in the third presumptive instance of IMNMwith dysphagia as the initial symptom. Given that isolated dysphagia in IMNM is atypical to the conventional symptoms in the late stage of the disease, it is critical for clinicians to have a high degree of suspicion for IMNM due to the aggressive nature of the disease and its refractoriness to treatment. Additionally, this case also highlights an atypical autoantibody, PL-7, being positive in an IMNM patient who presents with dysphagia as an initial symptom.

## Introduction

Idiopathic inflammatory myopathies (IIMs), which include dermatomyositis, polymyositis, overlap syndrome with myositis, inclusion body myositis (IBM), and immune-mediated necrotizing myopathy (IMNM), are rare autoimmune disorders that primarily affect skeletal muscle [1]. IMNM is a progressive disease that accounts for 19% of all inflammatory myopathies [2].

Dysphagia ensues in 20%-35% of IMNM patients [3]. One retrospective study examined 32 patients diagnosed with myopathies who presented with dysphagia as the initial or most prominent symptom [4]. The study identified five IMNM patients with dysphagia as the prominent symptom and one IMNM patient with dysphagia as the initial symptom [4]. Importantly, the study identified that patients who had dysphagia as the initial symptom demonstrated a longer time to diagnosis compared to patients who had more diffuse symptoms (36 vs. 24 months) [4].

Although various myopathies have been documented to be associated with dysphagia, there are limited studies of IMNM presenting with isolated or prominent dysphagia earlier in the clinical course. This atypical presentation may lead to the underdiagnosis of a fatal disease if left untreated. Here we present the third presumptive reported case of IMNM, with dysphagia as the initial symptom, followed by proximal muscle weakness. However, to the best of our knowledge, this is the first reported case of PL-7, as opposed to typical anti-signal recognition particles (SRP) or HMG-CoA reductase antibodies, associated with IMNM presenting with isolated dysphagia.

The abstract of this article was previously presented as a poster at the American College of Gastroenterology Conference in Charlotte, North Carolina, on October 24, 2022.

## Case presentation

A 74-year-old man with a past medical history of coronary artery disease, hypertension, and hyperlipidemia presented to the emergency department (ED) with two to three weeks of intractable nausea, vomiting, and dysphagia for solids and liquids. Vital signs were stable. The patient had a normal neurological exam, including 2+ deep tendon reflexes bilaterally in the extremities, full strength, no cranial nerve deficits, as well as a negative abdominal, neck, and throat exam.

As seen in Table 1, initial labs displayed an aspartate aminotransferase (AST) of 188 U/L and an alanine transaminase (ALT) of 64 U/L with normal bilirubin. A computed tomography (CT) of the chest, abdomen, and pelvis was all normal and did not reveal any findings that could potentially cause dysphagia.

**Table 1:** Laboratory values upon admission

Laboratory test	Result	Reference range
Hemoglobin (g/dl)	12.7	13-17.7
White blood cell count (per mm^3^)	5	3.5-10.9
Segmented neutrophils (%)	90	42-80
Sodium (mEq/L)	139	135 - 145
Potassium (mEq/L)	3.9	3.4 - 5.3
Calcium (mEq/L)	11.1	8.5 - 10.5
Magnesium (mg/dl)	2.1	1.4- 2.5
Phosphorous (mg/dl)	3.8	2.1- 4.3
Blood Urea (mg/dl)	18	3 - 29
Creatinine (mg/dl)	0.7	0.5 – 1.4
Total bilirubin (mg/dl)	1.4	0.0 – 1.2
Thyroid-stimulating hormone (uIU/mL)	3.5	<0.400 – 4.500
Free thyroxine (ng/dL)	1.2	0.8 – 1.80
Troponin (mg/dl)	18	3 - 29
Alanine aminotransferase (IU/litre)	64	0 - 60
Aspartate aminotransferase (IU/litre)	188	0 - 46

An esophagram showed moderate to severe tertiary contraction, no mass or stricture, and a 13-mm barium tablet passed without difficulty. Esophagogastroduodenoscopy (EGD) revealed a spastic lower esophageal sphincter, suggesting possible achalasia. Botox injections provided no significant relief. Manometry was recommended on an outpatient basis but never completed.

He then developed symmetrical proximal motor weakness four to five weeks later, which prompted further diagnostic testing. Laboratory evaluation demonstrated an elevated creatine kinase (CK) level of 6,357 U/L and an aldolase level of 43.4 U/L. Serology revealed positive PL-7 autoantibodies (Abs), but negative JO-1, PL-12, KU, MI-2, EJ, anti-SRP, anti-smooth muscle, and anti-mitochondrial Abs. A muscle biopsy was negative for endomysial inflammation or major histocompatibility complex (MHC) class I sarcolemmal upregulation. The diagnosis of IMNM was suspected.

A percutaneous endoscopic gastrostomy feeding (PEG) tube was placed as an alternative route of nutrition. He was started on steroids, and an outpatient rheumatology follow-up was arranged. He, unfortunately, passed away a month later due to complications from an unrelated coronavirus disease 2019 (COVID-19) infection.

## Discussion

Idiopathic inflammatory myopathies (IIMs) are all associated with muscle weakness, increased CK levels, disease-specific histopathological findings, and myopathic features on electromyography (EMG) [5]. The typical presentation of IMNM involves rapidly progressive symmetric proximal muscle weakness, markedly elevated CK levels, and often significant functional impairment [5]. Interstitial lung disease, respiratory dysfunction, hoarseness, cardiac complications, skin involvement, or extraocular features may also occur in IMNM [1, 6].

Dysphagia is known to occur in IIMs late in the clinical course due to the immune-mediated involvement of skeletal muscle affecting the oropharyngeal, laryngeal, and esophageal musculature, resulting in uncoordinated swallowing [7]. Dysphagia is estimated to develop in 10%-73% of IIM patients but has been reported most often in IBM and malignancy-associated dermatomyositis [7]. While about a third of patients with IMNM experience dysphagia in their clinical course, it is found that patients with idiopathic IMNM are more likely to have dysphagia in general [3].

Several Abs have been characteristically found in IMNM, including anti-3-hydroxy-3-methylglutaryl-coenzyme, A reductase (anti-HMGCR), and anti-signal recognition particle (anti-SRP) Abs [8]. Though the patient was negative for anti-SRP Abs and the anti-HMGCR Ab was not tested for in our patient, these Abs and the presence of myositis-specific Abs (MSA/MSAs) are not required for diagnosis [9]. Our patient also had no known risk factors associated with IMNM, including statin exposure, connective tissue disease (CTD), cancer, or human immunodeficiency virus (HIV) infection [3]. Diagnosis is further established by muscle biopsy or clinical criteria, including proximal muscle weakness and elevated CK [6]. Histopathology of IIMs demonstrates an inflammatory exudate, whereas IMNM is characterized by prominent muscle fiber necrosis without a significant lymphocytic inflammatory infiltrate [2]. While the patient's muscle biopsy did not specify any necrosis, it is possible that the sample was insufficient to determine evidence of active necrosis. The lack of necrosis on the biopsy was also probably due to obtaining the biopsy from a muscle with no active necrosis.

With IMNM and other inflammatory myositis diseases, there are no required criteria or a set number of criteria that a patient must meet in order to be diagnosed. The presumptive diagnosis of IMNM was sufficiently established given the presence of an MSA and the demonstration of most of the clinical features. IMNM is largely a clinically based diagnosis, with the presentation including the subacute development of symmetrical muscle weakness and fatigue, particularly in the proximal muscles, and labs supporting skeletal muscle inflammation [10]. Our patient had an elevation in ALT, AST, CK, and aldolase, indicating muscle enzyme involvement. Our patient was also tested for a multitude of Abs and was found to have positive PL-7 Abs, which is an MSA, which provides further support for our patient being diagnosed with inflammatory myositis [9]. The PL-7 Ab has been documented to be associated with the anti-synthetase syndrome, which is a rare autoimmune disease that is characterized by interstitial lung disease and/or inflammatory myositis, but the patient did not meet the diagnostic criteria for this diagnosis [11]. He lacked any evidence of interstitial lung disease or Raynaud’s phenomenon, which is commonly associated with PL-7 Abs and anti-synthetase syndrome [9]. The PL-7 Ab has also not been found to have a significant association with dysphagia. Moreover, the more commonly highlighted Abs linked to an increased risk of dysphagia include NXP2, FHL-1, SAE, HMGCR, NT5c1A, SRP, TIF1y, OJ, and some myositis-specific or associated autoantibodies in general [12]. Additionally, the PL-7 Ab, which is a subtype of anti-aminoacyl-tRNA synthetase (ARS) Abs, has been reported to be found in only 2%-5% of IIM patients [13]. This case highlights the unusual occurrence of dysphagia in a patient who is positive for PL-7 Abs, as well as the rarity of an IMNM patient being PL-7 Ab positive. To date, there have been no reported cases of IMNM with positive PL-7 Abs alone.

Dysphagia may require further evaluation, including swallow studies, contrast esophagrams, esophagogastroduodenoscopy (EGD), or esophageal manometry [4]. While achalasia is an important differential diagnosis to rule out with manometry, achalasia is unlikely to present with the rest of the symptoms the patient had aside from dysphagia. Additionally, the patient was admitted to a hospital where inpatient manometry could not be performed. Unfortunately, the patient did not complete the outpatient manometry testing. It is important to note that myositis patients presenting with dysphagia or muscle weakness may be misdiagnosed with amyotrophic lateral sclerosis, limb-girdle muscular dystrophy, or sarcoid myopathy, which may also exhibit increased CK levels with bulbar dysfunction [7, 14]. To prevent a delay in diagnosis, it is essential to include IMNM in the differential diagnosis of myositis and dysphagia in presenting patients.

Though there are minimal randomized trials regarding the treatment of IMNM, the treatment is similar to that of IIMs, which involve immunomodulatory therapy. First-line treatment involves corticosteroids, including high-dose prednisone, pulsed intravenous (IV) methylprednisone, or even IVIG [5]. Rituximab, azathioprine, and methotrexate may be used as second-line agents [5]. However, the presence of dysphagia complicates the management plan. Patients may need dietary modifications, swallowing therapy, or additional methods of adequate nutrition, such as a PEG tube or a nasogastric tube [4]. With dysphagia refractory to treatment, surgical intervention, including cricopharyngeal myotomy and esophageal balloon dilatation, may be necessary [4]. Esophageal balloon dilation tears the muscle fibers, which results in weakened and stretched skeletal muscle and, thus, improvement of dysphagia symptoms [15]. Early recognition of IMNM and aggressive treatment may lead to more favorable outcomes [3].

## Conclusions

In conclusion, IMNM is a rapidly progressive disease that requires a high degree of suspicion among clinicians, given its potential evolution to severe functional impairment. With few cases reported in the literature of dysphagia occurring as an initial or prominent symptom in IMNM patients, this case adds to the literature by reporting a patient who experiences dysphagia as an initial symptom. Additionally, the case highlights the unusual occurrence of dysphagia in a patient who is positive for PL-7 Abs, as well as the rarity of an IMNM patient being PL-7 Ab positive, as this Ab is uncommonly seen in IMNM or dysphagia patients. Atypical presentations or rapid advancement of isolated dysphagia should prompt clinicians to consider the diagnosis of IMNM within the differential. Though diagnostic criteria have been established for IMNM, there is a significant need for further research regarding the establishment of optimal treatment strategies. Future studies are required to standardize treatment algorithms as IMNM with isolated dysphagia remains an uncommon presentation.
